# Social Origins of Rhythm? Synchrony and Temporal Regularity in Human Vocalization

**DOI:** 10.1371/journal.pone.0080402

**Published:** 2013-11-29

**Authors:** Daniel L. Bowling, Christian T. Herbst, W. Tecumseh Fitch

**Affiliations:** 1 Department of Cognitive Biology, University of Vienna, Vienna, Austria; ARC Centre of Excellence in Cognition and its Disorders (CCD), Australia

## Abstract

Humans have a capacity to perceive and synchronize with rhythms. This is unusual in that only a minority of other species exhibit similar behavior. Study of synchronizing species (particularly anurans and insects) suggests that simultaneous signal production by different individuals may play a critical role in the development of regular temporal signaling. Accordingly, we investigated the link between simultaneous signal production and temporal regularity in our own species. Specifically, we asked whether inter-individual synchronization of a behavior that is typically irregular in time, speech, could lead to evenly-paced or “isochronous” temporal patterns. Participants read nonsense phrases aloud with and without partners, and we found that synchronous reading resulted in greater regularity of durational intervals between words. Comparison of same-gender pairings showed that males and females were able to synchronize their temporal speech patterns with equal skill. These results demonstrate that the shared goal of synchronization can lead to the development of temporal regularity in vocalizations, suggesting that the origins of musical rhythm may lie in cooperative social interaction rather than in sexual selection.

## Introduction

A variety of animals that produce acoustic or visual signals in groups exhibit collective patterns of temporal signal interactions [1], [2]. Well-studied examples are found in arthropods, anurans, birds, and mammals (including at least one non-human ape [3]). Among the most precise of these interactions are *synchrony* and *alternation* [1]. These phenomena occur in species where the signals produced by individuals are fundamentally periodic, comprising regular temporal intervals between components [1], [2]. In the synchronous Southeast Asian firefly (*Pteroptyx malaccae*), for example, congregating males flash every 0.6 seconds in nearly perfect phase with their neighbors for hours on end [2], [4]. Other examples of precise temporal patterns predicated on rhythmic signaling include the advertisement songs of some cicadas [5], the chorusing behavior of various orthoptera [6], [7], bouts of male-male alternation in a variety of New World frogs [2], [8], and synchronous claw waving in male fiddler crabs [9].

In humans, the temporal characteristics of signal production have been studied most extensively in the context of speech. Much contemporary research originated in the idea that languages can be categorized according to which elements in the speech signal (e.g., syllables or stresses) occur at isochronous (i.e., evenly-paced) intervals [10], [11]. However, empirical work has generally failed to find evidence of consistent isochrony in normal speech [12], [13], finding instead that perceptual distinctions in linguistic rhythm are founded on variation in factors such as syllable complexity and vowel reduction [13], [14]. The absence of isochrony in speech contrasts with the central role that isochrony plays in most music in the form of a steady beat, or *tactus*. Although a few musical styles do not use tactus-based rhythm [15], some form of steady beat constitutes a fundamental organizing principle in a huge variety of traditions from around the world [16]. This fundamental difference in temporal organization between isochrony in music and temporal irregularity in speech raises important questions for theories that propose a common origin for these behaviors [17]–[26]. Did the putative ancestral musical protolanguage comprise regular, isochronous rhythms? If so, why has this feature disappeared in normal speech? If not, why has it developed in music? Although the first, evolutionary, question is difficult to answer, clues to the latter two questions come from considering the social context in which regular temporal patterns occur. Cross-cultural comparisons of musical styles in our own species [15], [27] and cross-species comparisons of rhythmic signaling (see above and cf. [2], [25]) both suggest a potentially critical role of *simultaneous* signal production by different individuals in the development of isochronous temporal patterns. A possible reason for this connection is that isochrony makes the behavior of others predictable, thereby facilitating precise temporal coordination by a group.

To test this hypothesis, we examined the effect of simultaneous signal production on the development of temporal regularity in human vocalization. Specifically, we asked whether a requirement of inter-individual synchrony can transform a behavior that is typically irregular in time, i.e. speech, into one that exhibits regular temporal patterns. Using a paradigm in which participants read aloud with and without partners, we show that synchrony results in greater regularity of durational intervals between words, and that males and females are able to synchronize their temporal speech patterns with equal skill. These results demonstrate that the shared goal of synchrony can lead to the development of temporal regularity in human vocalizations, providing evidence for the possible origins of musical rhythm in social interaction.

## Methods

### Ethics statement

The experiment reported in this article was conducted in accordance with Austrian law and the policies of the University of Vienna. According to the Austrian Universities Act 2002, the appointment of ethics committees is required only for medical universities engaged in clinical tests, the application of new medical methods, and/or applied medical research on human subjects. Accordingly, ethical approval was not required for the present study. Nevertheless, all participants gave written informed consent and were aware that they could withdraw from the experiment at any time without further consequences. All data was stored anonymously.

Speech was recorded in two contrasting conditions to facilitate comparison of timing in the context of solo and simultaneous production. In the “alone” condition, we recorded participants reading aloud by themselves. In the “social” condition, participants read aloud together in pairs, and were instructed to synchronize their voices. The reading material consisted of three short sentences and was the same across conditions and experimental sessions. Each sentence comprised six pseudowords made from 1–3 syllables randomly selected from the set: [ba], [bi], and [bo]. The number of one-, two-, and three-syllable words in each sentence was held constant, but their positions were randomized. Each sentence thus comprised 11 syllables, with 10 inter-syllable intervals (5 within words and 5 between words), e.g., “ba bobo bi babobo babo bobi”. The use of nonsensical as opposed to real sentences served two purposes. First, it minimized the temporal influence of semantics and/or culturally conditioned aspects of word pronunciation. Second, it simplified the identification of syllable onsets and thus facilitated accurate measurements of temporal structure (see below).

40 participants (aged 18–45, *mean  = 24* years; 20 male) were recruited using job advertisement websites in Vienna, Austria. All participants were native speakers of German and had less than three years of experience (*mean*  = 0.54) in activities that might train synchronization abilities (e.g. playing a musical instrument/singing or taking dance classes). The latter restriction was aimed at reducing the possibility that familiarity with isochrony-based synchronization techniques in music would bias the strategy used by participants in the present task. To examine possible gender differences in synchronization ability, participants were run in pairs of the same gender. Pair members were strangers, with no relationship prior to participation.

Each session consisted of the members of a pair alternately participating in the alone condition, followed by simultaneous participation in the social condition, followed by repeated participation in the alone condition. Repetition of the alone condition after the social condition allowed determination of whether differences in speech timing might be explained by practice/familiarity with the sentences [28]. At the beginning of the first alone condition, participants received written instructions (see Text S1) that included correct syllable pronunciations and the directive to read each sentence as naturally as possible. At the beginning of the social condition, participants were instructed to read the same sentences while attempting to “match their voices together in time”. No mention was made of music, rhythmicity, regular timing, or keeping a beat. The quality of a pair's synchronization attempt was ensured by requiring participants to complete three recordings of each sentence with sub-threshold synchronization scores (see “sync score” described below). Participants were informed of this requirement, and told that they would receive feedback after each recording on whether or not they had synchronized successfully. A maximum of 20 attempts was permitted for each sentence. If participants failed to complete three recordings with sub-threshold synchronization scores within this allotment, their data were excluded from further temporal analysis. The rationale for exclusion was that poor synchronization limited our ability to quantify the effect of synchronization on temporal regularity. On the basis of pilot work with 5 participant pairs, it was estimated that ∼5–7 recordings per sentence would be required to successfully complete the synchronization task in the social condition. The number of recordings per sentence required in the alone conditions was thus set at seven to promote consistency in the number of recordings between conditions. In both conditions, the beginning of each recording was signaled by a one second tone at 440 Hz after which the participant(s) could begin reading at any time.

All recordings were made in an anechoic chamber at the University of Vienna. In the alone condition, one participant waited outside this room while their partner was recorded inside and was thus unable to hear their partner's speech. In the social condition, two sound-isolating booths located inside the room were utilized to facilitate acoustic separation of the recorded signals (the recording set-up is diagrammed in Figure S1). Each booth was equipped with a head-mounted microphone (DPA 4061; http://www.dpamicrophones.com/en/products.aspx?c=Item&category=128&item=24039#specifications) for speech recording, as well as headphones (Sennheiser HD201) that allowed participants to hear their own voice, the voice of their partner, the voice of the experimenter, and the tones that signaled the beginnings of recordings (these computer-generated signals were supplied to the headphones via a Mackie 402VLZ3 mixer). Temporal alignment of speech recordings from the two booths was ensured by simultaneous recording onto the left and right tracks of a stereo .wav file using a Zoom H4n Recorder (sampling rate  = 44.1 kHz, bit depth  = 16).

Assessments of temporal structure in the speech recordings were based on syllable onsets, defined as rapid changes in intensity associated with the onset of voicing immediately following the plosive release of [b] sounds. Syllable onsets were identified using a two-step procedure. First, the recorded waveform was processed by a computer algorithm that identified candidates on the basis of local maxima in the first derivative of an intensity contour (this algorithm is described in Text S2). Second, these candidates were subjected to manual confirmation by visual inspection using a graphic depiction of the waveform. Algorithm output and visual inspection were in agreement for 98.3% of all recorded syllables. The remaining 1.7% were adjusted to accord with visual inspection using a custom graphical user interface. This process permitted rapid identification of syllable onsets times immediately following each recording, which in turn allowed rapid calculation of the synchronization score used to assess synchrony in the social condition. This “sync score” was defined as the mean difference (in ms) in corresponding syllable onset times between participants – lower scores thus indicated more precise synchrony. In the pilot work mentioned above, we found that all 5 participant pairs achieved sync scores below 40 ms within 5–7 attempts (see [29] for similar results). Accordingly, a threshold sync score of 40 ms was chosen to define successful synchronization.

Temporal regularity of the recorded speech signals was assessed by calculating the coefficient of variation (CV) for interval durations between adjacent syllable onsets in each recording. CV was preferred over standard deviation because it also takes into account the average duration of inter-onset intervals, which varied considerably across recordings. Due to a significant difference in the duration of intervals that occurred between words (e.g., between “ba” and “bo” in “ba bobi”, *mean*  = 536 ms) compared to the those that occurred within words (e.g., between “bo” and “bi” in “ba bobi”, *mean*  = 276 ms), *t*(39) = 14.87, *p* = 1.17×10^−17^, CVs were calculated separately for these two interval types. Each recording thus yielded two measurements, a CV for intervals between adjacent syllable onsets across words, and a different CV for intervals between syllable onsets within words. Average CVs for each subject were calculated using the three sub-threshold recordings of each sentence from the social condition, and recordings 5–7 of each sentence in the alone conditions. The decision to use recordings 5–7 was made to make the data from the alone and social conditions as comparable as possible in terms of the number of previous recordings. However, using recordings 3–5 instead produced nearly identical results (see Figure S2).

While large CVs clearly indicate greater variability, a pattern of intervals with a large CV may still exhibit considerable regularity in time. For example, the pattern of interval durations 1-½-½-1-½-½-1, is more variable than 1-1-1-1-1-1-1, but remains regular in that its component intervals are related by small integer ratios. A further metric of temporal structure, called rhythmic division (RD), was developed and applied to the data to test for this type of temporal regularity. For a given set of intervals, RD was defined as the proportion of intervals related to the longest interval in the set to within ±5% of one of the following ratios: 1/8, 1/4, 1/3, 1/2, or 1/1. If intervals are related by these ratios, RD will be high, if not, RD will be low. RD calculations were made on the same recordings as the CV calculations.

Finally, the effect of gender on ability to synchronize was examined by comparing performance between female and male pairs on the synchronization task in the social condition. Two measures of performance were used: (1) the total number of recordings required by a pair to complete the synchronization task across all three sentences (minimum  = 9, maximum  = 60); and (2) a pair's mean sync score calculated across their 9 sub-threshold recordings.

## Results

Of the 20 pairs that participated in this study, 18 completed the synchronization task with relative ease, requiring a median of 5 recordings per sentence to achieve sub-threshold synchronization (*range* = 3–14). The remaining two pairs failed to achieve three sub-threshold recordings of each sentence. One of these pairs came close, missing a third sub-threshold recording only on the first sentence. The other pair only achieved two sub-threshold recordings across all three sentences.

**Figure 1:**
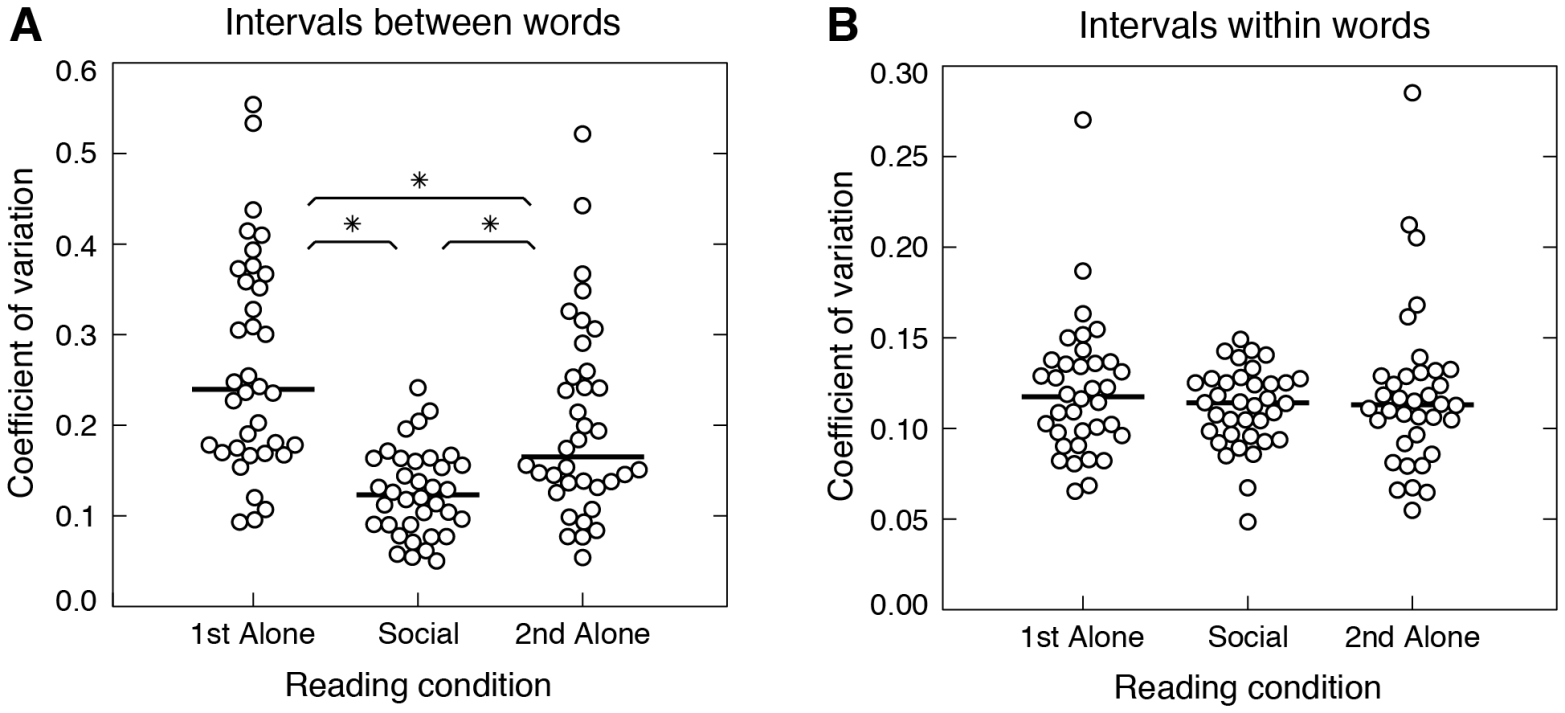
Coefficient of variation results. (A) Distributions of average CVs for intervals between words in the first alone, social, and second alone conditions. Each circle represents the mean CV for one subject in a given condition (*N* = 36). Horizontal black bars represent medians. Asterisks indicate statistical significance (*p*<0.0001). (B) Distributions of CVs for intervals within words, same format as A.

The results of the temporal regularity analysis for members of the 18 pairs that successfully completed the synchronization task are shown in Figure 1. CVs of intervals between words in the first alone condition (*median* = 0.24, *range* = 0.093-0.55) were approximately two times larger than those in the social condition (*median* = 0.12, *range* = 0.05–0.24), Wilcoxon *W* = 19, *Z* = −4.93, *p* = 8.09×10^−7^ (Figure 1A). A comparable difference was observed between the social condition and second alone condition (*median* = 0.17, *range* = 0.054–0.52), Wilcoxon *W* = 68, *Z* = −4.16, *p* = 3.14×10^−5^, showing that practice/familiarity with the sentences alone cannot explain the difference between conditions. However, the fact that a significant difference was also observed between the first and second alone conditions, Wilcoxon *W* = 39, *Z* = −4.62, *p* = 3.86×10^−7^, suggests that practice, and/or lingering influence of timing in the social condition, had some effect. No significant differences were observed with respect to intervals within words, which were less variable overall, with median CVs between 0.11 and 0.12 across conditions (Figure 1B).

**Figure 2:**
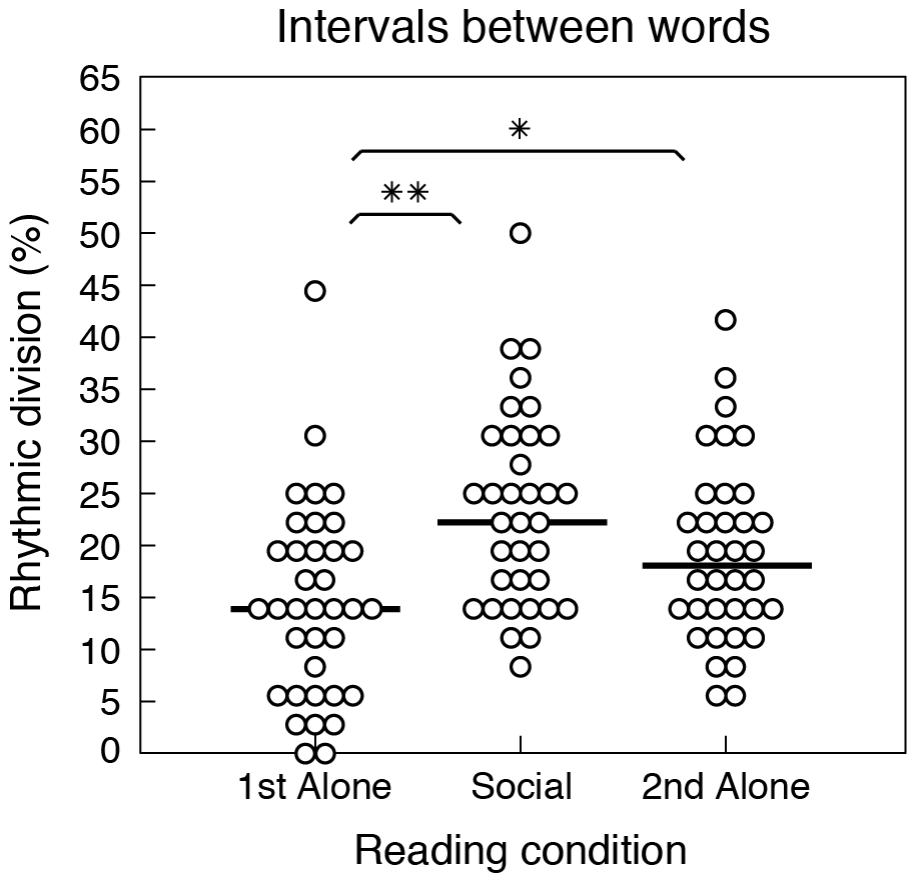
Rhythmic division results. Distributions of RD scores for intervals between words in the first alone, social, and second alone conditions. Each circle represents the mean RD score for one subject in a given condition (*N* = 36). Horizontal black bars represent medians. Asterisks indicate statistical significance (**p<0.001, *p<0.05). RD scores for intervals within words are not shown because the CVs of these intervals were not found to vary between conditions (see Figure 1B).

The results of the rhythmic division analysis are shown in Figure 2. RD scores in the social condition (*median* = 22%, *range* = 8–50%) were significantly higher than those in the first alone condition (*median* = 14%, *range* = 0–44%; Wilcoxon *W* = 73.5, *Z* = −3.57, *p* = 0.00036) and higher than those in the second alone condition (*median* = 18%, *range* = 6–42%), although this second difference did not attain significance (Wilcoxon *W* = 166.5, *Z* = −1.83, *p* = 0.068). There was thus a greater tendency for intervals to be related by small integer ratios in the social condition, compared to alone conditions, indicating that regular divisions of time were more prevalent in synchronized compared to solo speech. Comparison of the first and second alone conditions showed significantly higher RD scores in the second alone condition, Wilcoxon *W* = 162.5, *Z* = −2.12, *p* = 0.035, confirming the increase in regularity suggested by lower CVs (see above). Together the CV and RD data provide clear evidence that synchrony between individuals increases the temporal regularity of speech.

**Figure 3:**
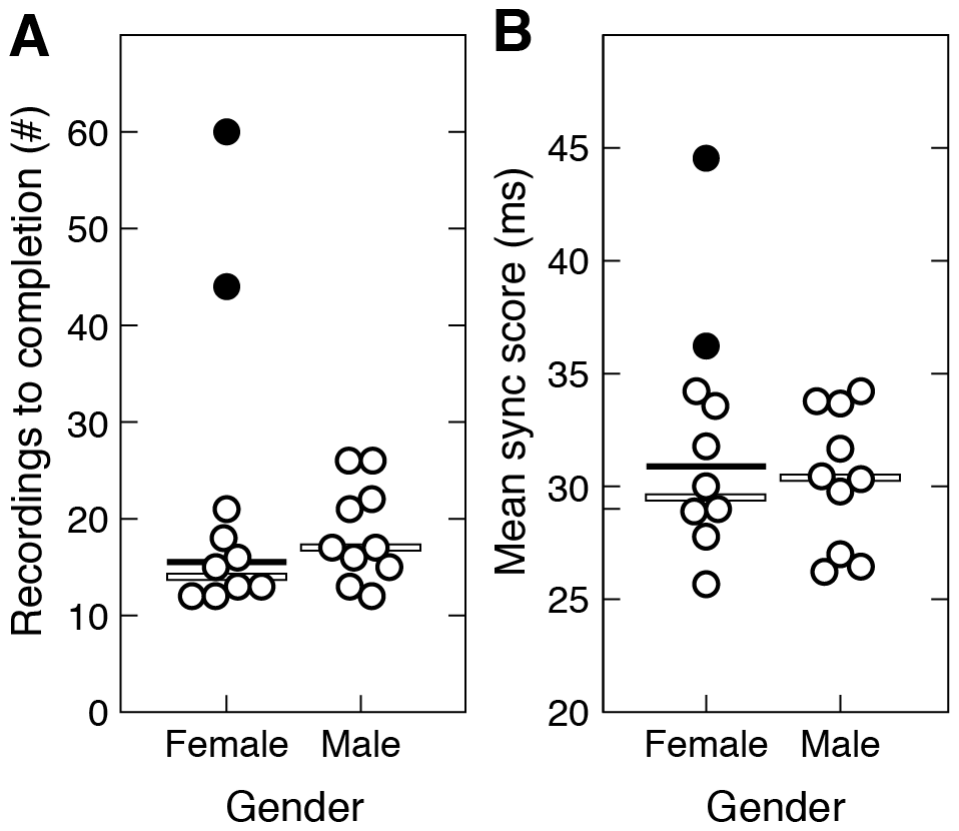
Gender analysis results. (A) Distributions of the number of recordings required by female and male pairs to complete three sub-threshold recordings of each sentence in the social condition. Each circle represents number of trials for a single participant pair (*N* = 20). Black circles represent data from the two pairs that failed to complete the synchronization task. Horizontal white bars represent medians excluding data from the failed pairs; horizontal black bars represent medians including data from the failed pairs. (B) Mean sync scores on sub-threshold recordings, same format as A. Mean sync scores for the failed pairs were calculated on the three recordings of each sentence with the lowest sync scores.

The results of the gender analysis are shown in Figure 3. No significant difference between male and female pairs was observed in either the number of recordings required to complete the synchronization task (Mann-Whitney *U* = 58.5, *p* = 0.13; Figure 3A), or mean sync score across sub-threshold recordings (Mann-Whitney *U* = 73, *p* = 0.83; Figure 3B). Even with the inclusion of data from the two participant pairs that failed to successfully complete the synchronization task (both of which were female-female; see black circles in Figure 3), the differences between genders on these comparisons remained insignificant (number of recordings to completion: Mann-Whitney *U* = 42.5, *p* = 0.6; mean sync score: Mann-Whitney *U* = 44, *p* = 0.68).

Although the focus of this work is on the temporal characteristics of vocalization, the data are also well-suited to questions about the effect of synchronization on the fundamental frequency (another important aspect of vocalization). Accordingly, a brief analysis of fundamental frequencies in speech from the alone and social conditions was made. The results show that, like temporal intervals between words, frequency intervals between syllables were also significantly less variable in the social compared to alone conditions (see Figure S3 and Text S3 for further details).

## Discussion

The results presented here show that inter-individual synchrony increases the temporal regularity of speech. Regularity increased significantly for intervals between words but not for intervals within words. The increased regularity of intervals between words could not be explained as an effect of practice/familiarity with the sentences alone (see Figure 1A), or deviation from more variable but nonetheless regular temporal patterns (see Figure 2). This suggests that increased regularity of intervals between words arose specifically to facilitate synchronization, presumably by allowing participants to accurately predict the timing of their partner's speech and coordinate their behavior accordingly.

The absence of an effect of synchrony on intervals within words likely reflects the lower overall variability of intersyllabic intervals within words (cf. Figure 1A and 1B). The syllables [ba], [bi], and [bo] all pair the consonant [b] with a monophthong, and thus leave little room for temporal variation in production. Had more complex and variable syllables been included (e.g., [bao] or [stra]), variability for within word intervals would likely have been greater and an influence of synchrony on their regularity might possibly have been observed. However, the decision to use simple syllables with similar structure benefitted the present experiment by easing the identification of syllable onsets (and thus simplifying temporal measurements) and avoiding variations in the perception of speech timing that occur with more complex syllables [30], [31].

It is important to note that the increase in temporal regularity observed in the present experiment was not a necessary consequence of the requirement for vocal synchrony. While some degree of predictability between individuals must be maintained for synchronization, there are multiple ways in which such predictability can be achieved. For example, rather than predicting syllable onsets on the basis of a regular temporal interval, one could predict them on the basis of their partner's timing on the previous recording. If your partner's timing was more irregular than yours, this strategy will lead to a decrease in temporal regularity. Two participants in the present experiment (from different pairs) demonstrated that this strategy, though rare, actually occurs. These participants successfully synchronized by matching their partner's irregular timing, resulting in significant decreases in the regularity of their speech (see Figure S4 and Text S4 for further detail). These data clearly show that increased temporal regularity is not a necessary consequence of synchronization. However, decreases in regularity were rare and found for only 2/36 successful synchronizers. The vast majority of participants used strategies that resulted in increased temporal regularity, presumably because if a regular temporal interval can be implicitly agreed upon, the reduction in memory requirements makes the task considerably easier.

One remaining issue concerns whether the increased temporal regularity observed here reflects a capability of humans that would develop in the absence of any previous experience with synchronization in musical context. Despite the fact that participants were selected on the basis of their lack of musical training, all adult humans can be expected to have had at least some experience with rhythmic synchronization, whether singing in a group or dancing to music. Thus, it remains possible that such experience biased the strategy used by participants in the present experiment. Although this possibility cannot be ruled out entirely, we emphasize that the present task was focused on speech and made no mention of rhythm or music. There are thus two reasons to doubt an explanation of the results based on musical experience: (1) the musical experience of the participants was limited; and (2) the task was not explicitly musical.

The present results are largely consistent with considerable work on synchronized speech by Cummins [28], [29], [32], [33]. Among other things, Cummins and his colleagues have demonstrated that people can synchronize their voices with relative ease at latencies comparable to those described here (i.e., <40 ms [29]), and that variability in the placement and duration of pauses between sentences is reduced in synchronized speech [32]. The principal contribution of the present work is an examination of the effect of speech synchronization on temporal regularity at a finer time-scale, comparable to the beat-by-beat regularity typically observed in music. We found that inter-individual vocal synchrony leads to increased temporal regularity at this level, suggesting that this type of social interaction provides a plausible origin for the regular rhythms so widely observed in music.

Why synchronize? In the present study, pressure to synchronize was provided by an artificial task requirement, but if the origins of rhythm lie in synchronous vocal interaction, where did the pressure to synchronize come from? One recent theory that places vocal synchrony at the origins of human rhythmic abilities proposes that a selective pressure on males to synchronize was originally supplied by migrating females whose decision to settle with a particular group of males was determined in part by the synchronicity of multi-male vocal displays [20], [23], [25]. According to this hypothesis, synchronous vocalizations could have influenced migrating females in at least three ways: (1) synchronous vocalizations can sum in amplitude resulting in higher-power composite signals that travel farther with greater intensity and thus have more potential to attract females (see Figure S5 and Text S5); (2) increased intensity may have served as an indication of the resource richness of the territory held by a male group; and (3) high quality synchrony may have also indicated something about the capacity of a particular group for cooperation, which may have had additional benefits in resource acquisition and territorial defense [20], [23], [25]. To the extent that these factors actually did affect the choice to settle by migrating females, there would have been sexual selection on males to develop vocal synchrony skills. The present results provide evidence both for and against this hypothesis. In support, the fact that pressure to synchronize does indeed give rise to increased temporal regularity provides evidence that synchronous vocal display is a plausible origin of the human capacity for isochronous signal production and entrainment. Against this hypothesis, our finding that males and females were equally skilled in achieving vocal synchrony casts doubt on the role of sexual selection as the (sole) selective force promoting synchronous display. One intriguing alternative possibility is that the pressure to synchronize resulted from a more general “cooperative urge” – a motivation to share experiences, activities, and emotions with others – that is so typical of both sexes in our species, and so unusual in the natural world [34]–[37].

## Supporting Information

Figure S1 **Experimental set-up.** (A) An overhead view of the recording chamber. Acoustic attenuation between the sound booths was assessed by playing white noise through a speaker placed in one booth (90 dB measured at 10 cm) and comparing the spectra of the signals recorded by the microphones in booth 1 and 2. This procedure was repeated twice with the speaker placed in either booth 1 or 2, and the recorded signals were averaged according to whether the microphone location was on the same or opposite side of the speaker. In this way it was determined that inter-booth attenuation was approximately −27 dB at 50 Hz, −38 dB at 100 Hz, −46 dB at 200 Hz, −59 dB at 500 Hz, and −65 dB at higher frequencies. (B) A schematic diagram showing the equipment used to make the speech recordings. Arrowheads indicate the direction of signal flow.
(TIF)

Figure S2 **Coefficient of variation results for alone recordings 3–5 instead of 5–7.** Format is same as Figure 1 in the main text. Using recordings 3–5 instead of 5–7 changed the median CV of intervals between words from 0.24 (range = 0.093–0.55) to 0.27 (range = 0.11–0.64) in the first alone condition, and from 0.17 (range = 0.054 = 0.52) to 0.17 (range = 0.06–0.49) in the second alone condition. All significant differences between the alone and social conditions reported in the main text were preserved (first alone vs. social: Wilcoxon *W* = 15, *Z* = −5, *p* = 5.85×10^−7^; second alone vs. social: Wilcoxon *W* = 59, *Z* = −4.31, p = 1.67×10^−5^; first alone vs. second alone: Wilcoxon *W* = 56, *Z* = −4.35, p = 1.35×10^−5^). Again, no significant differences for intervals within words were observed between conditions.
(TIF)

Figure S3 **Synchrony and fundamental frequency variability.** (A) Box plots showing standard deviations in the size of frequency intervals (in cents) between syllables from the same recordings used to assess temporal regularity in the alone and social conditions. Horizontal red bars depict medians, boxes depict inter-quartile range (IQR), dashed-lines depict data within 1.5× IQR of the 25^th^ and 75^th^ percentiles, and crosses show data points lying outside this range (*p<0.0001). (B) Overlapping distributions showing the average frequency of occurrence of different interval sizes in the same recordings examined in (A). Only distributions for the first alone (red) and social conditions (blue; purple shows overlap) are shown. Histogram bin size  = 25 cents. See Text S3 for further discussion.
(TIF)

Figure S4 **Synchronization despite decreased temporal regularity.** (A) Same as Figure 1A but highlighting data from participants 33 and 14, both of which successfully synchronized with their partners despite significant decreases in the temporal regularity of their speech (**p<0.0125 *p<0.05). (B) Plots showing the strategy used by participants 33 and 14 to synchronize with their partners in the social condition. Top panels show “mean difference scores” (see Text S4) obtained from comparing the participant's timing on the specified recording with their partner's timing on the previous recording. Bottom panels show the participant's CVs for same recordings. All data pertain to the intervals between words from recordings of sentence 1. Dashed blue represent the average mean difference score across recordings for successfully synchronizing participants. Dashed green lines represent the average CV across the same recordings. Red data points indicate the recordings where sub-threshold synchrony was achieved. See Text S4 for further discussion.
(TIF)

Figure S5 **Amplitude summation in synchronous vocalization.** Box plots showing the mean amplitudes (root mean square) of the left, left+right, and right tracks of all recordings with sub-threshold sync scores from the social condition. Horizontal red bars depict medians, boxes depict inter-quartile range (IQR), dashed-lines depict data within 1.5× IQR of the 25^th^ and 75^th^ percentiles, and crosses show data points lying outside this range (*p<0.001). See Text S5 for further discussion.
(TIF)

Text S1 **Written instructions.** The text provided to participants at the beginning of the experiment.
(DOCX)

Text S2 **Details of the syllable onset identification procedure.** Further description of the computer algorithm and visual inspection procedure used to identify syllable onset times.
(DOCX)

Text S3 **Synchrony and fundamental frequency variability.** Explanation of the method used to assess fundamental frequency variability and discussion of the data presented in Figure S3.
(DOCX)

Text S4 **Synchronization despite decreased temporal regularity.** Explanation of the method used to calculate the “mean difference score” in Figure S4B and further discussion of how synchronization can occur despite decreased temporal regularity.
(DOCX)

Text S5 **Amplitude summation in synchronous vocalization.** Explanation of the method used to assess amplitude summation and discussion of the data presented in Figure S5.
(DOCX)
