# Supplementary figures and images for: Social Origins of Rhythm? Synchrony and Temporal Regularity in Human Vocalization
Source: PLoS One. 2013 Nov 29;8(11):e80402. doi: 10.1371/journal.pone.0080402 (PMC3843660; doi:10.1371/journal.pone.0080402)

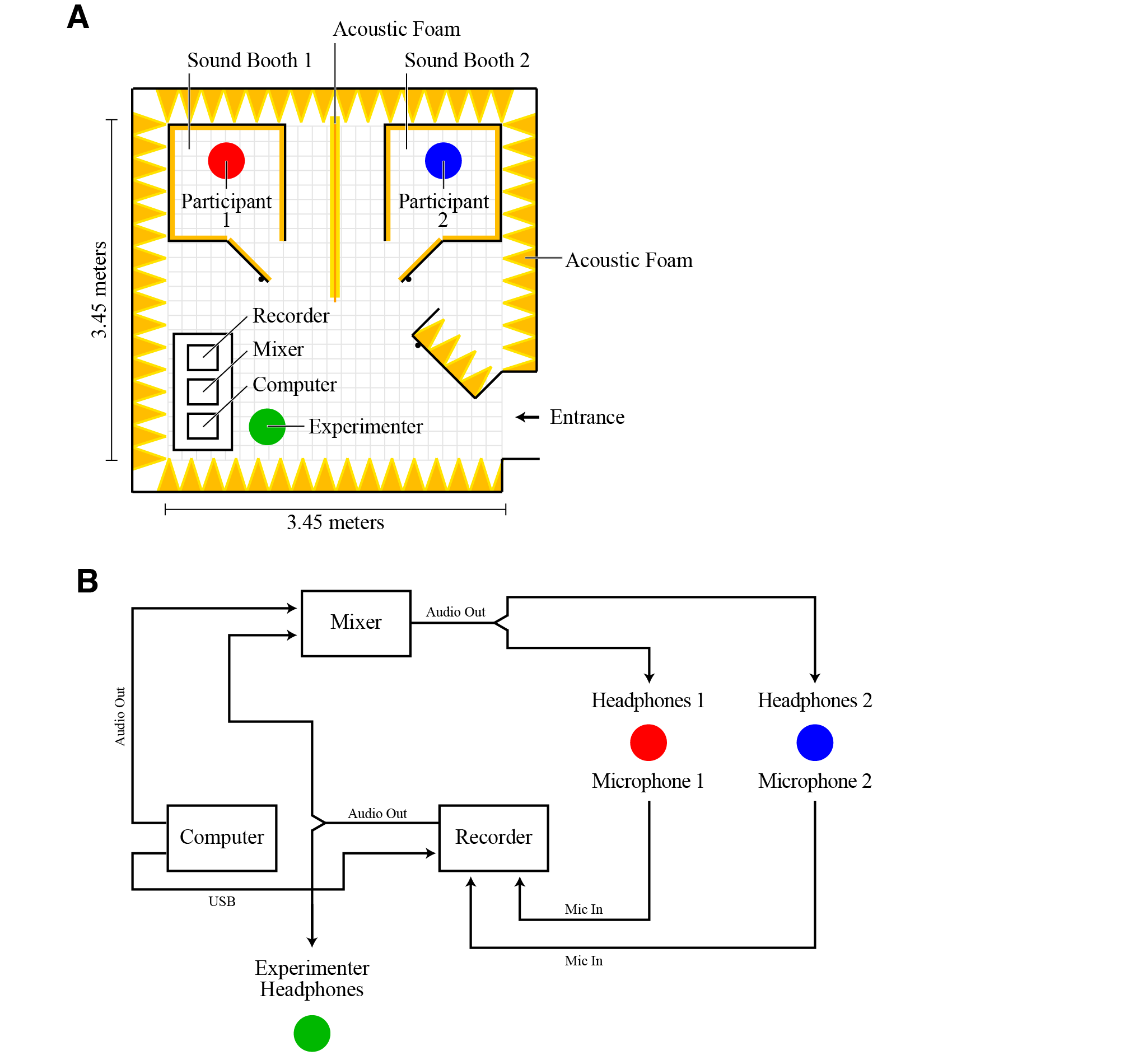

Supplement: Figure S1 — Experimental set-up. (A) An overhead view of the recording chamber. Acoustic attenuation between the sound booths was assessed by playing white noise through a speaker placed in one booth (90 dB measured at 10 cm) and comparing the spectra of the signals recorded by the microphones in booth 1 and 2. This procedure was repeated twice with the speaker placed in either booth 1 or 2, and the recorded signals were averaged according to whether the microphone location was on the same or opposite side of the speaker. In this way it was determined that inter-booth attenuation was approximately −27 dB at 50 Hz, −38 dB at 100 Hz, −46 dB at 200 Hz, −59 dB at 500 Hz, and −65 dB at higher frequencies. (B) A schematic diagram showing the equipment used to make the speech recordings. Arrowheads indicate the direction of signal flow. (TIF) [file pone.0080402.s001.tif]

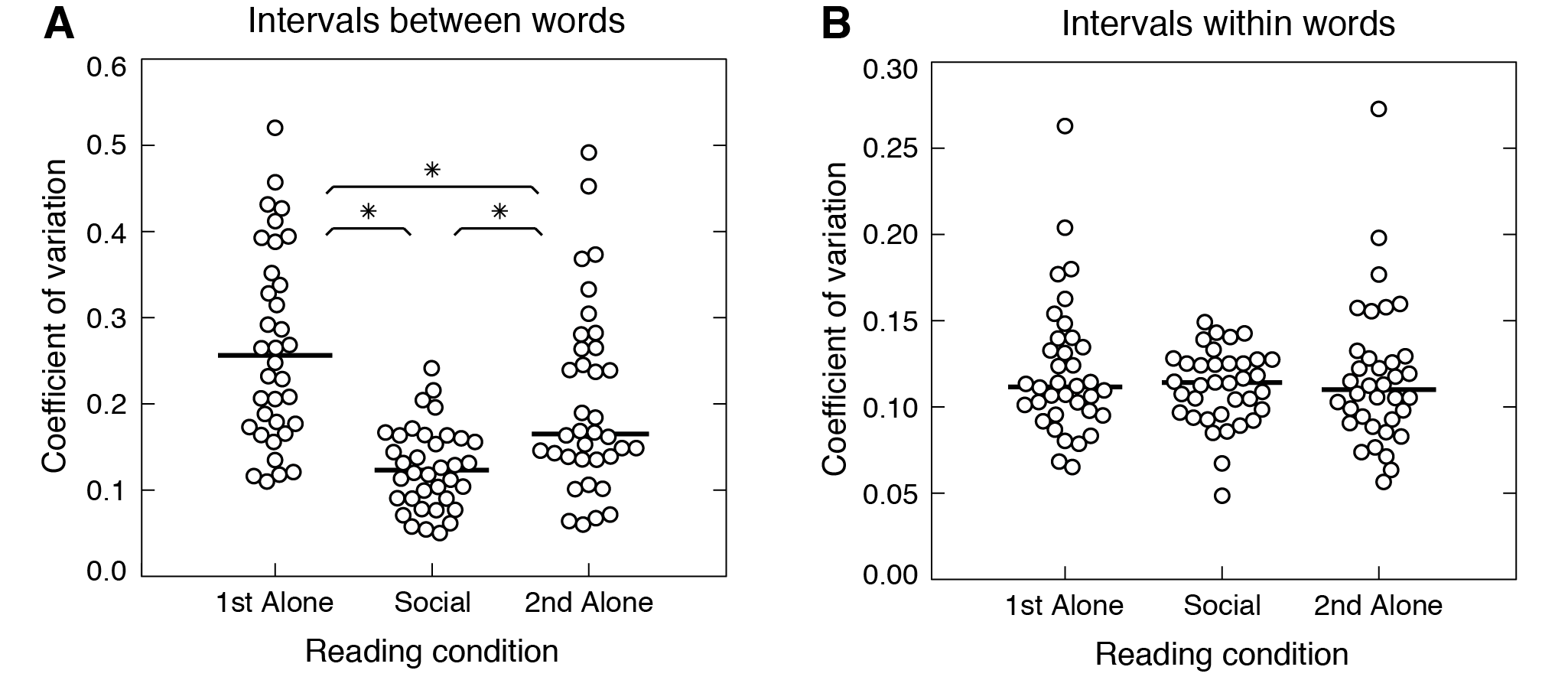

Supplement: Figure S2 — Coefficient of variation results for alone recordings 3–5 instead of 5–7. Format is same as Figure 1 in the main text. Using recordings 3–5 instead of 5–7 changed the median CV of intervals between words from 0.24 (range = 0.093–0.55) to 0.27 (range = 0.11–0.64) in the first alone condition, and from 0.17 (range = 0.054 = 0.52) to 0.17 (range = 0.06–0.49) in the second alone condition. All significant differences between the alone and social conditions reported in the main text were preserved (first alone vs. social: Wilcoxon W = 15, Z = −5, p = 5.85×10^−7^; second alone vs. social: Wilcoxon W = 59, Z = −4.31, p = 1.67×10^−5^; first alone vs. second alone: Wilcoxon W = 56, Z = −4.35, p = 1.35×10^−5^). Again, no significant differences for intervals within words were observed between conditions. (TIF) [file pone.0080402.s002.tif]

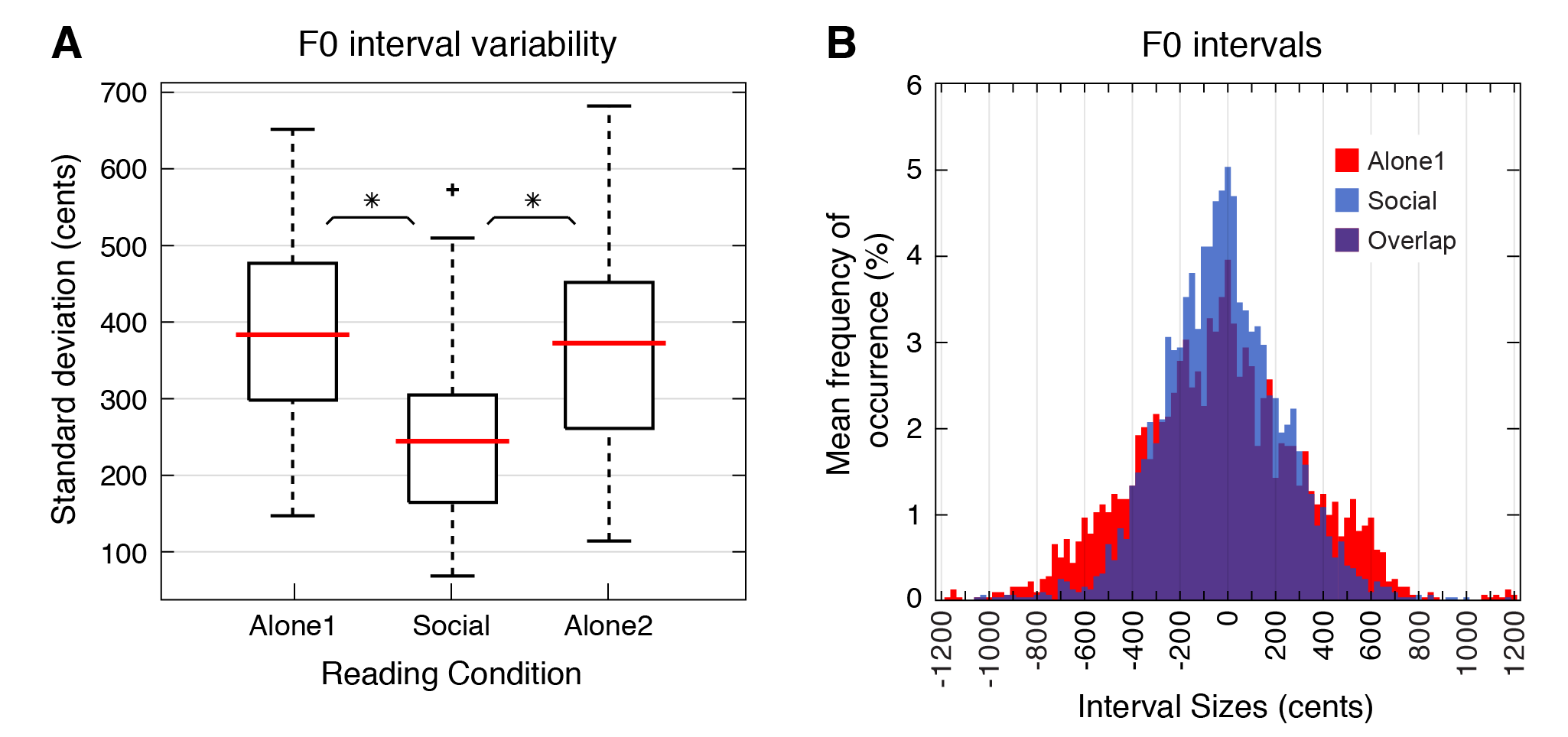

Supplement: Figure S3 — Synchrony and fundamental frequency variability. (A) Box plots showing standard deviations in the size of frequency intervals (in cents) between syllables from the same recordings used to assess temporal regularity in the alone and social conditions. Horizontal red bars depict medians, boxes depict inter-quartile range (IQR), dashed-lines depict data within 1.5× IQR of the 25th and 75th percentiles, and crosses show data points lying outside this range (*p<0.0001). (B) Overlapping distributions showing the average frequency of occurrence of different interval sizes in the same recordings examined in (A). Only distributions for the first alone (red) and social conditions (blue; purple shows overlap) are shown. Histogram bin size = 25 cents. See Text S3 for further discussion. (TIF) [file pone.0080402.s003.tif]

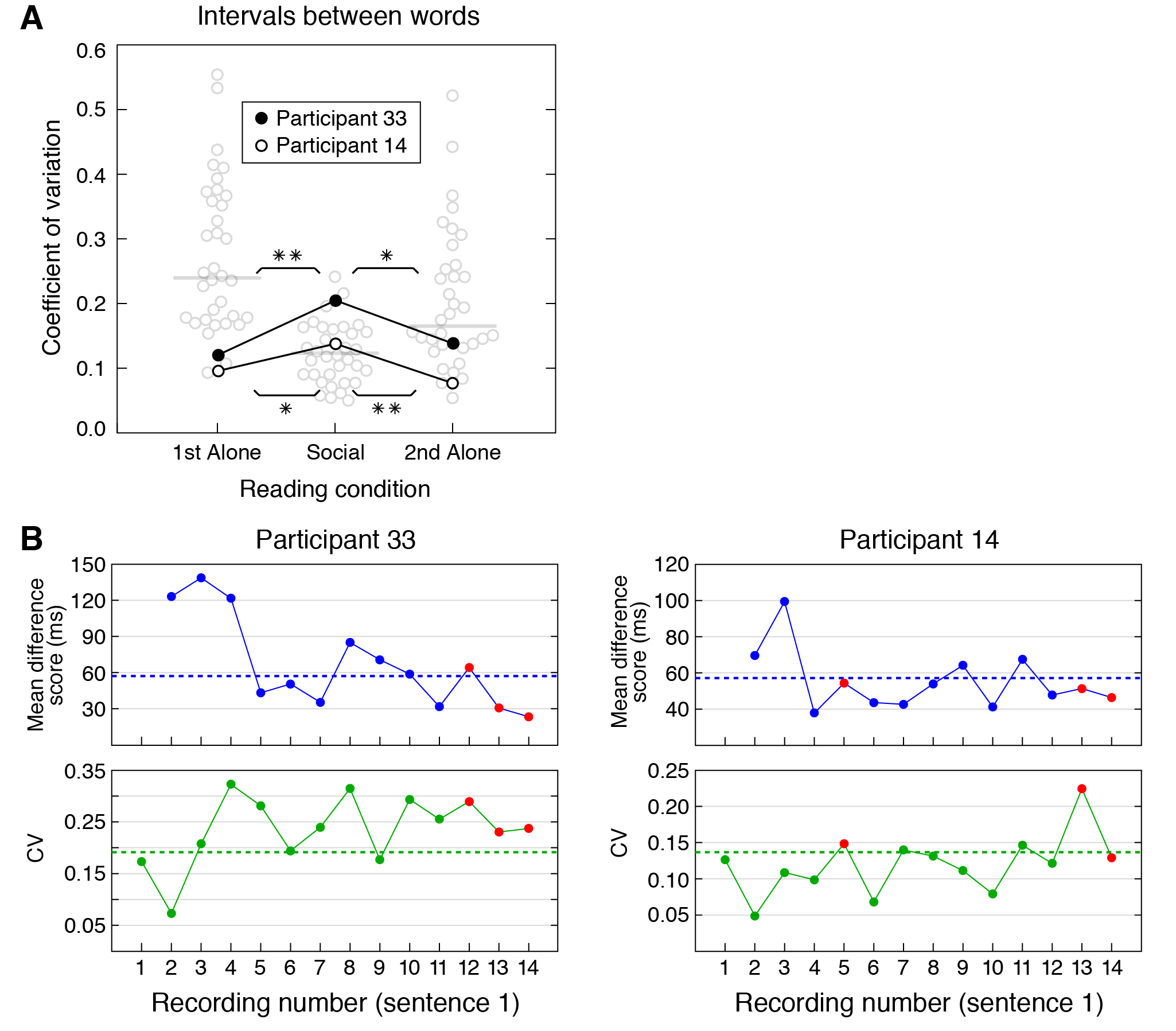

Supplement: Figure S4 — Synchronization despite decreased temporal regularity. (A) Same as Figure 1A but highlighting data from participants 33 and 14, both of which successfully synchronized with their partners despite significant decreases in the temporal regularity of their speech (**p<0.0125 *p<0.05). (B) Plots showing the strategy used by participants 33 and 14 to synchronize with their partners in the social condition. Top panels show “mean difference scores” (see Text S4) obtained from comparing the participant's timing on the specified recording with their partner's timing on the previous recording. Bottom panels show the participant's CVs for same recordings. All data pertain to the intervals between words from recordings of sentence 1. Dashed blue represent the average mean difference score across recordings for successfully synchronizing participants. Dashed green lines represent the average CV across the same recordings. Red data points indicate the recordings where sub-threshold synchrony was achieved. See Text S4 for further discussion. (TIF) [file pone.0080402.s004.tif]

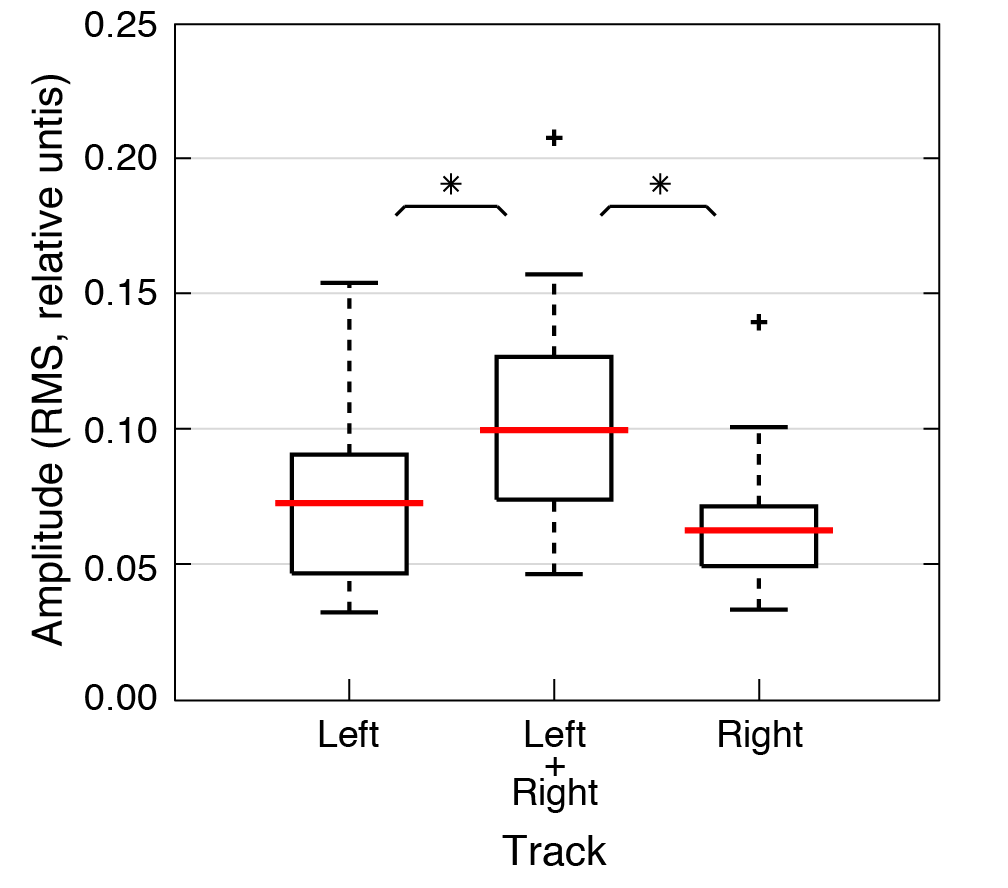

Supplement: Figure S5 — Amplitude summation in synchronous vocalization. Box plots showing the mean amplitudes (root mean square) of the left, left+right, and right tracks of all recordings with sub-threshold sync scores from the social condition. Horizontal red bars depict medians, boxes depict inter-quartile range (IQR), dashed-lines depict data within 1.5× IQR of the 25th and 75th percentiles, and crosses show data points lying outside this range (*p<0.001). See Text S5 for further discussion. (TIF) [file pone.0080402.s005.tif]
