# Supplementary material for: Social Origins of Rhythm? Synchrony and Temporal Regularity in Human Vocalization
Source: PLoS One. 2013 Nov 29;8(11):e80402. doi: 10.1371/journal.pone.0080402 (PMC3843660; doi:10.1371/journal.pone.0080402)
Supplement: Text S1 — Written instructions. The text provided to participants at the beginning of the experiment. (DOCX) [file pone.0080402.s006.docx]

**Text S1. Written Instructions.**

At the beginning of the experiment, participants were provided with the following instructions printed on a piece of paper:

In this experiment you will be asked to read nonsensical sentences out loud. For example:

**BABOBI BIBA BO BIBA BO BOBI**

Please read this sentence as if it were a real sentence with real words.

Every sentence is made from the syllables BA, BO, and BI. **BA** is pronounced as in *baden*, **BO** as in *bohne,* and **BI** as in *biene.*

Practice the example sentence until you are comfortable. Tell the experimenter when you are ready to continue.

After the participant informed the experimenter they were ready to continue, they were asked to flip the paper over to see the sentences they would be reading, and to begin reading the first sentence after the tone. The sentences used in this experiment were:

1. **BABOBI BIBA BO BIBA BO BOBI**
2. **BOBA BA BIBO BI BIBOBO BABI**
3. **BOBO BABA BA BABI BA BIBOBI**

See the main text (Methods, second paragraph) for an explanation of how these sentences were constructed.
