# Supplementary material for: Social Origins of Rhythm? Synchrony and Temporal Regularity in Human Vocalization
Source: PLoS One. 2013 Nov 29;8(11):e80402. doi: 10.1371/journal.pone.0080402 (PMC3843660; doi:10.1371/journal.pone.0080402)
Supplement: Text S2 — Details of the syllable onset identification procedure. Further description of the computer algorithm and visual inspection procedure used to identify syllable onset times. (DOCX) [file pone.0080402.s007.docx]

**Text S2*.*** **Details of the syllable onset identification procedure.**

The onset of each syllable was defined as the time-point where the rising slope of the intensity contour associated with the plosive release of the consonant [b] reached a local maximum. The algorithm designed to identify syllable onsets operated in five steps: (1) the recorded signal was rectified and a moving average was applied to generate an intensity contour (window length =50 ms, time step =1 ms); (2) the intensity contour was low-pass filtered (3^rd^ order Butterworth, cut-off =50 Hz) to create a smoothed intensity contour free of high frequency fluctuations unrelated to syllable onsets; (3) the first and second derivatives of the smoothed intensity contour were calculated, and positive-to-negative zero crossings in the second derivative were used to identify local maxima in the first derivative; (4) local maxima that were not the greatest within a 200 ms window (centered) were excluded; and (5), the 11 remaining greatest local maxima were returned as syllable onset candidates for visual inspection.

In the visual inspection phase of syllable onset identification, the waveform of the recorded signal, the smoothed intensity contour, its first derivative, and the 11 syllable onset candidates were plotted on the computer monitor. Syllable onsets could then be adjusted to other first derivative maxima using the mouse. Adjustments were made when an algorithmically identified candidate did not correspond to the plosive release of a [b] sound. Failure of the algorithm typically occurred when participants either prevoiced their [b] sounds or inserted a pause between plosive release and vowel onset. After completion of visual inspection, the times of syllable onset candidates originally identified by the algorithm, as well as those determined through visual inspection were recorded (resolution = 1 ms).
