# Supplementary material for: Social Origins of Rhythm? Synchrony and Temporal Regularity in Human Vocalization
Source: PLoS One. 2013 Nov 29;8(11):e80402. doi: 10.1371/journal.pone.0080402 (PMC3843660; doi:10.1371/journal.pone.0080402)
Supplement: Text S3 — Synchrony and fundamental frequency variability. Explanation of the method used to assess fundamental frequency variability and discussion of the data presented in Figure S3. (DOCX) [file pone.0080402.s008.docx]

**Text S3. Synchrony and fundamental frequency variability**

Fundamental frequency (F0) variability was assessed by comparing the standard deviation of F0 differences between syllables in the alone and social conditions. For each syllable, an F0 value was calculated 50 milliseconds after onset using Praat’s [38] built in “To Pitch” algorithm (pitch floor =75 Hz, pitch ceiling = 500 Hz). For 3.2% of syllables, Praat’s algorithm could not identify an F0 value at this time, in which case the next nearest time with an identifiable F0 value was used instead (on average, this next nearest time was ~40 milliseconds away). After each syllable in a recording had been assigned an F0 value, the size of the frequency difference (or *interval*) between each pair of adjacent syllables was calculated in cents (a logarithmic unit of interval size; 100 cents is equal to an equally tempered semitone). The standard deviation of these intervals was then calculated as a measure of F0 variability for that recording. For each subject that successfully completed the synchronization task in the social condition (N=36), these standard deviations were averaged for recordings 5-7 in the alone conditions, and the recordings with sub-threshold sync scores in the social condition. The resulting distributions are shown in Figure S3A. Standard deviations were significantly lower in the social condition (median =244 cents, range =69-773) than in the first alone (median =383 cents, range =147-652; Wilcoxon *W* =56, *Z* =−4.35, *p* =1.35x10^-5^) and second alone conditions (median =372, range =114-682; Wilcoxon *W* =68, *Z* =−4.16, *p* =3.14x10^-5^). ­­No significant difference was observed between the first and second alone conditions (Wilcoxon *W* =278, *Z* =−0.86, *p* =0.39). Figure S3B shows the distributions from which the standard deviations in Figure S3A were derived (for the first alone and social conditions).

The increased regularity of frequency intervals in synchronized speech is of particular interest because frequency, like time, is organized into regular intervals in music but not speech. These results thus provide further evidence that synchrony pushes vocalization in a “musical” direction.

**References**

1. Boersma P, Weenink D (2013) Praat: doing phonetics by computer [Computer program]. Version 5.3.21, retrieved 5 July 2012 from <http://www.praat.org/>
