# Supplementary material for: Social Origins of Rhythm? Synchrony and Temporal Regularity in Human Vocalization
Source: PLoS One. 2013 Nov 29;8(11):e80402. doi: 10.1371/journal.pone.0080402 (PMC3843660; doi:10.1371/journal.pone.0080402)
Supplement: Text S4 — Synchronization despite decreased temporal regularity. Explanation of the method used to calculate the “mean difference score” in Figure S4B and further discussion of how synchronization can occur despite decreased temporal regularity. (DOCX) [file pone.0080402.s009.docx]

**Text S4. Synchronization despite decreased temporal regularity.**

If a participant is attempting to synchronize with their partner by matching the irregular timing their partner exhibited on the previous recording, two things should be true. First, the difference in timing between the participant’s present recording and their partner’s previous recording should be small (suggesting attempted matching), and second, the coefficient of variation (CV) of intervals in the participant’s present recording should be high (suggesting temporal irregularity). Given that a method for calculating the CV of interval durations has already been described (see Methods), all that is needed is a method for calculating the difference in timing between a participant’s present recording (*P1_n_*) and their partner’s previous recording (*P2_n-1_*). One such method is to take each pair of recordings, *P1_n_* and *P2_n-1_*, and (1) calculate the absolute difference between each pair of corresponding interval durations, and (2) calculate the mean of these differences. This will result in a “mean difference score” for each recording 2 through *k,* where *k* is the total number of recordings of a sentence made by a participant pair in the social condition.

Two participants in different pairs, #33 and #14, succeeded in synchronizing with their partners despite significant decreases in temporal regularity relative to the alone conditions (Figure S4A). The mean difference scores and CVs of the intervals between their words in recordings of sentence 1 from the social condition are shown in Figure S4B. Looking first at participant 33 (left), it is apparent that the recordings where sub-threshold synchrony was achieved (red dots) all occur when the mean difference score is relatively low (suggesting attempted matching) but the CVs are relatively high (suggesting temporal irregularity). Relative lowness and highness can be judged by the comparing the position of the red dots to the dashed blue and green lines, which represent the average mean difference score and average CV respectively (calculated for intervals between words in recordings of sentence 1 from the social condition across all successfully synchronizing participants). A similar but less pronounced pattern is apparent for participant 14 (right). These results thus suggest that participants 33 and 14 synchronized with their partners by matching the irregular timing their partners exhibited on previous recordings.
