# Supplementary material for: Social Origins of Rhythm? Synchrony and Temporal Regularity in Human Vocalization
Source: PLoS One. 2013 Nov 29;8(11):e80402. doi: 10.1371/journal.pone.0080402 (PMC3843660; doi:10.1371/journal.pone.0080402)
Supplement: Text S5 — Amplitude summation in synchronous vocalization. Explanation of the method used to assess amplitude summation and discussion of the data presented in Figure S5. (DOCX) [file pone.0080402.s010.docx]

**Text S5. Amplitude summation in synchronous vocalization**

The effect of synchrony on the amplitude of the resulting sound signal was examined by comparing the root mean square (RMS) amplitude of the left and right tracks of the wav files from recordings with sub-threshold sync scores in the social condition, with the RMS amplitude of their summation (left + right). As expected, the amplitude of the left + right tracks (median =0.1, range =0.047-0.208) was always larger than that of the left (median =0.073, range =0.033-0.154) or right tracks (median =0.063, range =0.034-0.14) considered separately, resulting in significant differences across subjects (N=36), Wilcoxon *W* =0, *Z* =−3.72, *p* =0.000196, for both left vs. left + right, and right vs. left + right comparisons.

It is important to note, however, that the method of amplitude summation used here (adding together two digitally-represented acoustic waveforms with a computer program) differs in important ways from the kind of amplitude summation that can be expected to occur between synchronized vocalizations in the air. The primary reason is that computer summation bypasses environmental variables that affect how two sounds combine (e.g., the position of the vocalizers with respect to one another, the direction they are facing, the location of the receiver, the presence of other atmospheric disturbances, and the reflective/absorptive characteristics of other objects in the sound field). Thus, the differences in amplitude between individual and synchronized vocalizations shown in Figure S5 are likely to be higher than those that would be observed in the field.
